# MRI before biopsy correlates with depth of invasion corrected for shrinkage rate of the histopathological specimen in tongue carcinoma

**DOI:** 10.1038/s41598-021-00398-0

**Published:** 2021-10-25

**Authors:** Hiroyuki Harada, Hirofumi Tomioka, Hideaki Hirai, Takeshi Kuroshima, Yu Oikawa, Hitomi Nojima, Junichiro Sakamoto, Tohru Kurabayashi, Kou Kayamori, Tohru Ikeda

**Affiliations:** 1 Division of Oral Health Sciences, Department of Oral and Maxillofacial Surgery, Tokyo Medical and Dental University, 1-5-45 Yushima, Bunkyo-ku, Tokyo, 113-8549 Japan; 2 Division of Oral Health Sciences, Department of Oral and Maxillofacial Radiology, Tokyo Medical and Dental University, Tokyo, Japan; 3 Division of Oral Health Sciences, Department of Oral Pathology, Tokyo Medical and Dental University, Tokyo, Japan

**Keywords:** Cancer, Head and neck cancer, Oral cancer

## Abstract

The purpose of this study was to evaluate which radiological depth of invasion (r-DOI) measurement is the most concordant to clinical DOI (c-DOI) derived from correction for the shrinkage rate of the histopathological specimens. We retrospectively reviewed 128 patients with tongue carcinoma who had undergone glossectomy between 2006 and 2019. At first, the width shrinkage rate during formalin fixation and preparation process of histopathological specimens was evaluated. From the shrinking rates, a formula to calculate c-DOI from pathological DOI (p-DOI) was developed. The correlation between c-DOI and r-DOI was evaluated. The specimen shrinkage rate during the histopathological specimen preparation process was 10.3%. Based on that, we yielded the correct formula for c-DOI based on p-DOI and preparation shrinkage rate: c-DOI = p-DOI × 100/89.7. The regression equations for the association of c-DOI with r-DOI measured by ultrasound (n = 128), MRI before biopsy (n = 18), and MRI after biopsy (n = 110) were y = 1.12 * x + 0.21, y = 0.89 * x − 0.26, and y = 0.52 * x + 2.63, respectively, while the coefficients of determination were 0.664, 0.891, and 0.422, respectively. In conclusion, r-DOI using MRI before biopsy most strongly correlated with c-DOI.

## Introduction

Tongue carcinoma is the most common malignancy in the oral cavity. Since it is largely a surgically treated disease, it is crucial to determine the exact tumor location before surgery. In clinical practice, CT, MRI, and ultrasound, which can demonstrate the location of the tumor, are widely used in tongue squamous cell carcinoma (SCC). Since its introduction in the AJCC/UICC 8th edition of the TNM system of oral carcinoma^1,2^, depth of invasion (DOI) represents an important parameter in oral squamous cell carcinoma management because it can predict overall prognosis, risk of recurrence, and cervical lymph node metastases^3–7^. The radiological DOI (r-DOI) is frequently measured by CT, MRI, and ultrasound in tongue SCC. Previous studies have reported significant relationship between r-DOI measured on these modalities and pathological DOI (p-DOI) in the tongue SCC^8–12^. r-DOI differs from the tumor thickness (TT), and is defined as the distance from a virtual normal mucous membrane to the tumor’s deepest part on the image. However, tongue carcinoma often shows exophytic or remarkably ulcerous lesions, for which it is difficult to measure r-DOI using CT, MRI, and ultrasound^6,13,14^. Another problem is the deviation of 1.2–3 mm between r-DOI and p-DOI in tongue carcinoma^7–9^ because the width shrinkage rate during the preparation process of histopathological specimen was not considered. The purpose of this study was to evaluate which r-DOI measurement is the most concordant to clinical DOI (c-DOI) determined from p-DOI corrected for shrinkage.

## Results

### Shrinkage rate of specimens during the preparation process of histopathological specimen

The shrinkage rate at the maximum width diameter by formalin fixation (X → X′) was 1.6 ± 3.4% (Supplemental Table S1). The shrinkage caused by the preparation process of histopathological specimens from sectioning to final preparation (Y → Y′) was 8.8 ± 5.4%. Finally, the specimen shrinkage rate during the histopathological specimen preparation process was 100 − (100 − 1.6) × (100 − 8.8)/100 = 10.3 ± 6.3%. Hence, the correct formula to determine the c-DOI from p-DOI was as follows: c-DOI = p-DOI × 100/89.7.

### Relationships between r-DOI and c-DOI

Table 1 shows the summary of the patients’ data. The mean r-DOIs of ultrasound, MRI before biopsy, and MRI after biopsy were 7.0 mm, 8.6 mm, and 9.7 mm, respectively. The mean period from imaging to surgery in these groups was 21.0 days, 16.9 days, and 11.7 days, respectively.

**Table 1:** Summary of patients' data.

	Ultrasound (n = 128)	MRI before biopsy (n = 18)	MRI after biopsy (n = 110)
**Sex**			
Men	85	14	71
Women	43	4	39
**Age (year)**			
Mean	55.7	53.7	56.1
Range	21–86	26–86	21–86
**DOI (mm)**			
Mean	7.0	8.6	9.7
Range	2.4–17.9	3.2–24.0	3.5–23.2
**Period from imaging to surgery (day)**			
Mean	21.0	16.9	11.7
Range	4–46	4–60	5–28

*DOI* depth of invasion.

**Figure 1:**
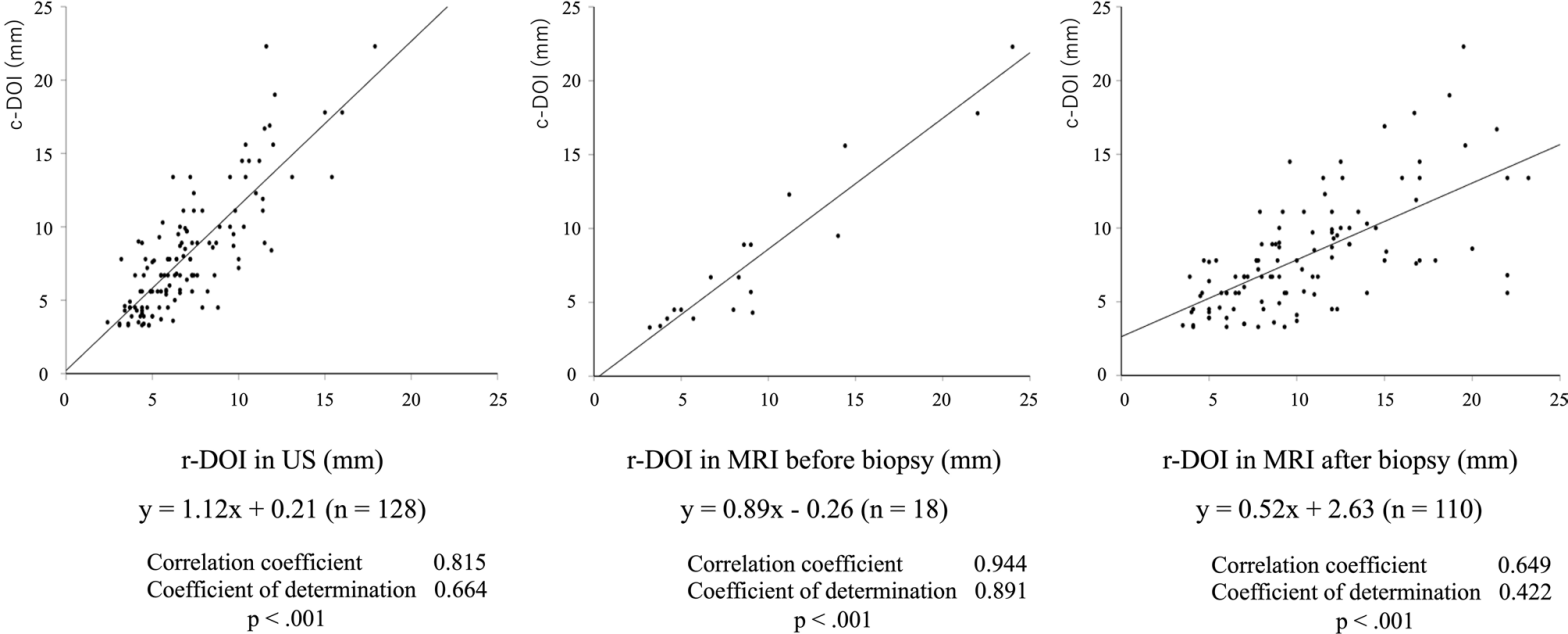
Relationship between c-DOI and each r-DOI. MRI before biopsy was the most concordant with c-DOI. r-DOI using MRI after biopsy overestimated DOI because of inflammatory reaction of the tongue muscles caused by biopsy. *c-DOI* clinical depth of invasion, *r-DOI* radiological depth of invasion.

We examined the correlation between c-DOI and r-DOI of ultrasound, MRI before biopsy, and MRI after biopsy. The corrected formula c-DOI = p-DOI × 100/89.7 was used. Figure 1 demonstrates the correlation between c-DOI and r-DOI. The regression equations for the association of r-DOI with ultrasound, MRI before biopsy, and MRI after biopsy were y = 1.12 * x + 0.21, y = 0.89 * x − 0.26, and y = 0.52 * x + 2.63, respectively, and they were all significant models. The correlation coefficients were 0.815, 0.944, and 0.649, respectively, and the coefficients of determination were 0.664, 0.891, and 0.422, respectively. MRI before biopsy was the most concordant with c-DOI, but still slightly overestimated it. Ultrasound slightly underestimated c-DOI. r-DOI using MRI after biopsy tended to overestimate c-DOI because of inflammatory reaction of tongue muscles to biopsy.

## Discussion

The shrinkage rate during preparation of histopathological specimens depends on specimen type and the preparation conditions, and varies from 14.7 to 47.3% in head and neck cancer^15–18^. Those previous studies reported on the shrinkage rate of mucosal longitudinal and/or transverse length. While surgical margins in head and neck cancer specimens easily shrink after formalin fixation, specimens were fixed by pins during formalin fixation in this study. No studies have clarified the width measured from the surface of mucosa to the deepest tumor site. This is the first report to evaluate the width shrinkage rate during preparation process of histopathological specimens fixed by pins. The pins fixed the specimen to reproduce an anatomical form, preventing shrinking of the mucous membrane, not compressed with weight of specimens in formalin. Here we showed that the shrinkage rate of tongue width was 10.3%. Therefore, c-DOI could be calculated according to the following formula; c-DOI = p-DOI × 100/89.7.

Of note, the shrinkage rate depends on the presence or absence of pins, formalin dipping time, paraffin infiltration process, room temperature, etc. Thus, the shrinkage rate may vary between facilities. To ensure consistency, in this study, all procedures were performed in constant conditions by a single pathologist.

There are many comparative studies between r-DOI and p-DOI using ultrasound^8,19^ or MRI^3,7,20,21^. Several studies stated that CT^22,23^ is also useful for evaluation of r-DOI; however, CT cannot evaluate r-DOI when the contrast is low, superficial lesions cannot be detected, and metal artifacts often disturb images of tumor location. Therefore, we used ultrasound and MRI. Yesuratnam et al.^24^ reported the correlation between tumor thickness on ultrasound and MRI with histopathologically determined TT of tongue carcinoma. Preoperative TT determined by US demonstrated higher correlation with pathological TT, compared with TT determined by MRI. However, there were several problems in this report^24^, including evaluating histopathological TT instead of p-DOI, inflammation due to biopsy as MRI was taken after biopsy, and ignorance of the shrinkage of specimens. Our study excluded cases of exophytic tumor and remarkably ulcerous lesions because measurement of r-DOI in these tumor types is unreliable. r-DOI on MRI was divided into “before biopsy” and “after incision biopsy”, and considering the width shrinkage rate during preparation of histopathological specimens.

Ultrasound has advantages as being an easy method and can easily assess even superficial tumors because of a sufficiently high contrast. Yet, the measurement of exophytic tumor and remarkably ulcerous lesions is difficult, and it is hard to insert the probe in case of a posterior tumor of the tongue.

The advantage of the MRI is that the image is acquired in the tongue’s resting position and that there is no upper limit of the measured value. The disadvantages include difficult detection of superficial tumors, influence of metal artifacts, and movement of the tongue. We used MRI for cases with p-DOI > 3 mm, because 3 mm is an MRI cutoff value of detectable lesions. Similarly, Baba et al. reported that the cutoff value of p-DOI for detectable lesion on MRI was 4 mm^25^. Therefore, the detection limit of p-DOI on the MRI is likely 3–4 mm.

Several studies have reported a significant relationship between r-DOI measured on MRI and p-DOI in the tongue SCC^3,7,20,21^. To the best of our knowledge, this is the first study on MRI divided into “before biopsy” and “after incision biopsy”. Our prior study focusing on the period between 2006 and 2015 showed that r-DOI using MRI before biopsy best correlated with p-DOI^26^. The present study expanded on this by including a larger patient number and considering the width shrinkage rate during the preparation process of histopathological specimen. Finally, similar results were obtained. It is desirable that the regression equation of c-DOI is y = x, which would indicate that c-DOI and r-DOI are equal. The regression equation of MRI before biopsy was y = 0.89 * x − 0.26, suggesting slight overestimation of c-DOI. This is probably due to reflection of stromal reaction around the tumor, such as lymphoplasmocytic infiltration. However, the coefficients of determination were very high (0.891), and r-DOI using MRI before biopsy was the most reliable. The regression equation of MRI after biopsy was y = 0.52 * x + 2.63, indicating severe overestimation of c-DOI because of inflammatory reaction of the tongue muscles caused by biopsy^4,27^. Biopsy may lead to edema or hemorrhage and subsequent overestimation of tumor size and invasion depth in MRI^4,24^. Especially in small tumors, if they are biopsied prior to imaging, the inflammation may affect the MRI interpretation. Further studies are necessary to determine how much the inflammatory reaction of biopsy spreads and how long an inflammatory reaction after the biopsy continues on MRI.

On the other hand, the regression equation for ultrasound was 1.12 * x + 0.21. The coefficient of determination was 0.664. This means slight underestimation of c-DOI and a wide distribution. These caused influencing to measure putting forward tongue, the period from the image to surgery was longest, and a border may not depict the boundary unclear such as high grade of histopathological pattern of invasion.

Our study had several limitations. First, the shrinkage rate was measured at the maximal width of surgical specimen, not of DOI of the tumor. However, measurement of DOI at the maximal cross-sectional area of the tumor before formalin fixation made making a slide at the same slice difficult. Second, the number of cases with MRI before biopsy was small (n = 18). We usually performed the biopsy at first presentation to operate earlier because MRI could not be taken immediately but there was a waiting time. Thus, patients were usually subjected to MRI after the biopsy in this study. However, the mean period from imaging to surgery in individuals in whom MRI was performed before biopsy was 16.9 days, which was longer than that in individuals in whom MRI was performed after biopsy (11.7 days). Because the long waiting time until operation would likely influence the treatment results, an early operation is desirable. Although it is difficult to improve the waiting time until the operation in many facilities, a higher number of cases in future studies would be required.

In conclusion, the specimen shrinkage rate during the histopathological specimen preparation process was 10.3%. After correcting p-DOI accordingly, r-DOI using MRI before biopsy most strongly correlated with c-DOI.

## Methods

### Patient material

**Figure 2:**
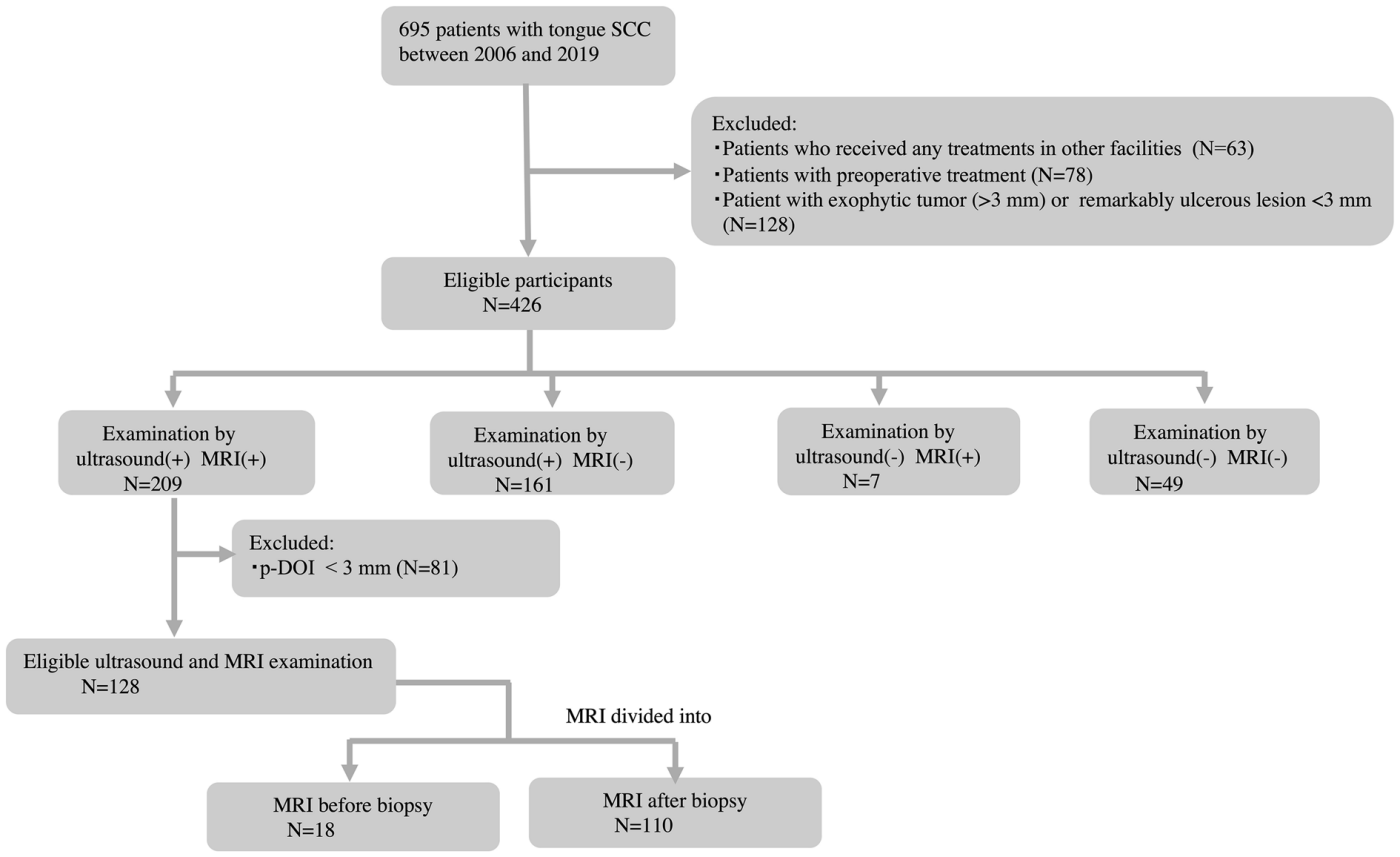
Flowchart of the inclusion and exclusion decision tree. Out of 695 patients, we excluded 63 patients who had received any treatment in other facilities, 78 patients who had undergone preoperative treatment at initial treatment, and 128 individuals who showed exophytic type more than 3 mm from the normal mucosal surface or those with ulceration deeper than 3 mm. There were 426 eligible participants. Out of these, both ultrasound and MRI was examined in 209 patients. Eighty-one cases with p-DOI < 3 mm were excluded because 3 mm was an MRI cutoff value of detectable lesions. Finally, 128 patients were enrolled on ultrasound and MRI. In addition, MRI was divided into “before biopsy” (N = 18) and “after biopsy” (N = 110). *p-DOI* pathological depth of invasion.

We retrospectively reviewed the medical records of 695 consecutive patients with tongue SCC who had undergone radical surgery between 2006 and 2019 (Fig. 2). Out of 695 consecutive patients, we excluded 63 patients who had received any treatments for tongue tumor in other facilities and 78 patients who underwent preoperative chemotherapy and/or radiotherapy at initial treatment. Given low reliability of r-DOI measurement in exophytic and remarkably ulcerous lesions by ultrasound and MRI, 128 cases that showed exophytic type more than 3 mm from the normal mucosal surface or cases with ulceration deeper than 3 mm from the normal mucosal surface were excluded. There were 426 eligible patients. Out of these, ultrasound and MRI were both available in 209 patients.

An early-stage oral tongue carcinoma was sometimes undetectable on MRI. We investigated the cut-off between detectable and undetectable lesions on MRI by receiver operating characteristic (ROC) analysis, and found a 3-mm threshold (sensitivity 80.6%, specificity 80.0%, and AUC 0.867, [Supplemental Fig. S1]). Therefore, we excluded 81 cases with p-DOI < 3 mm. Finally, 128 cases were enrolled on ultrasound and MRI. There were 85 men and 43 women, and their age ranged from 21 to 86 years (mean, 55.7 years). Macroscopic type of the tumor was superficial in 29 patients and endophytic in 99 patients. Intraoral ultrasound was performed before incisional biopsy, at first presentation. In our department, biopsy is basically performed at the time of first presentation, and imaging examinations, including MRI, are scheduled as early as possible. There were 110 cases that applied these criteria (1–32 days after biopsy, average 8.0 days). However, there were 18 cases in which consent for biopsy was not obtained at first presentation, and those patients underwent biopsy with consent after imaging examination including MRI. The Institutional Review Board of the Faculty of Dental Hospital of Tokyo Medical and Dental University approved this radiographic study, and written informed consent was obtained from all of the patients (approval No. D2015-600). The authors confirm that all experiments were conducted in accordance with the relevant guidelines and regulations.

### Shrinkage rate during the preparation process of histopathological specimens

**Figure 3:**
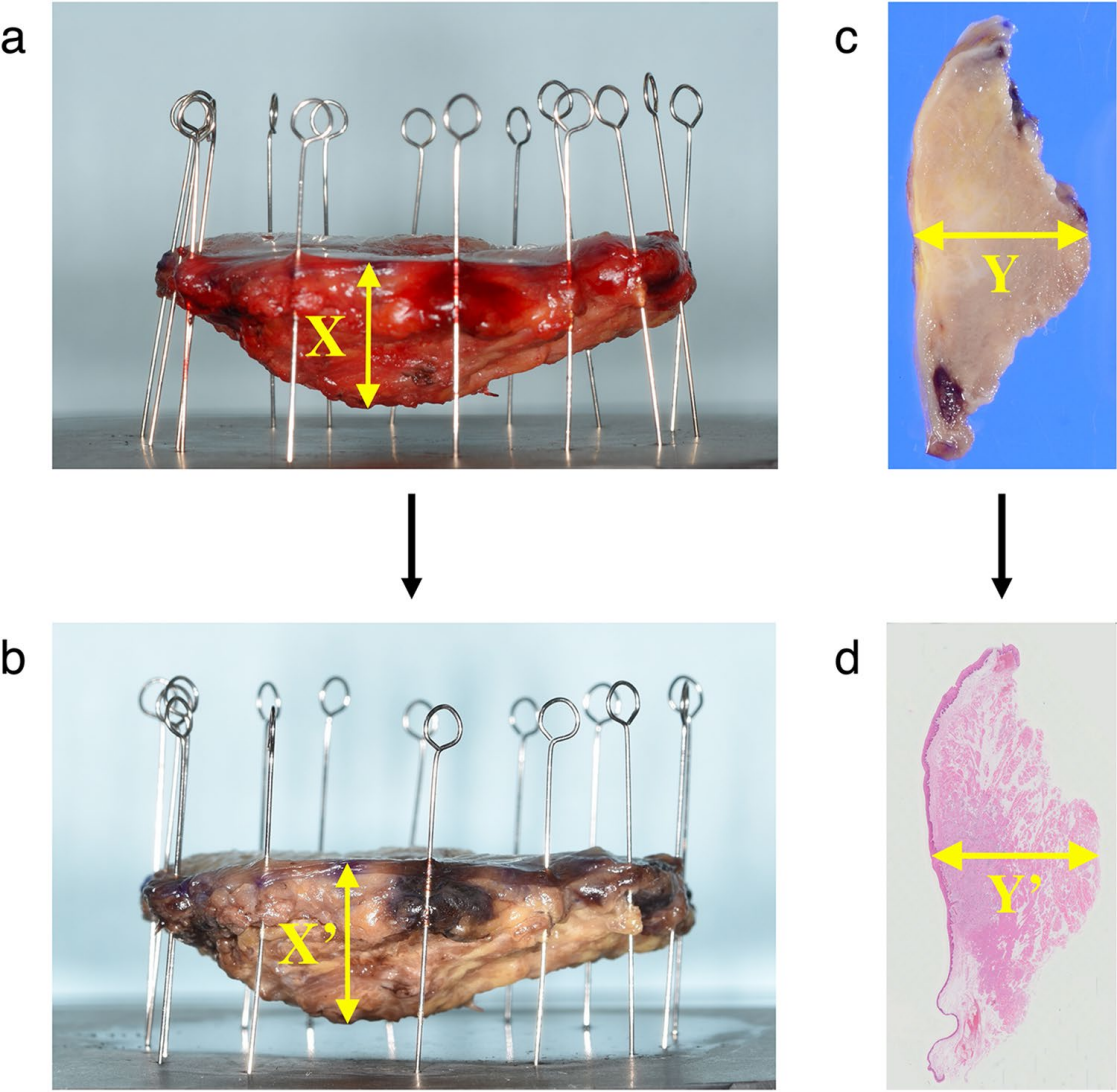
(**a**) Before formalin fixation and (**b**) after formalin fixation. The shrinkage rate at the maximum width by formalin fixation (a → b). The maximum width was measured (X [mm]) before formalin fixation. After fixation, the width was measured at the same site as X and determined as X′ (mm). (**c**) Sliced specimen and (**d**) slide specimen. The shrinkage caused by the preparation process of histopathological specimen from sectioning to final preparation (c → d). In the sliced specimen after formalin fixation, the maximum width of maximum cross-sectional area was measured (Y [mm]). On a glass slide, the same site as Y, Y′ (mm), was measured.

We evaluated the width shrinkage rate caused by formalin fixation (1) and preparation process of histopathological specimen from sectioning to final preparation (2). From 128 patients, specimens of 25 patients with sufficient data available were selected for the study. The specimens were fixed by pins to reproduce the preoperative anatomical structure (Fig. 3a) and fixed in 10% neutral buffered formalin for approximately 24 h. The maximal width was measured before (X [mm]) and after formalin fixation (X′ [mm]) (Fig. 3a,b). The shrinkage rate by formalin fixation was given as (X − X′)/X × 100 (%). Each specimen was sliced vertically from anterior to posterior at 5-mm intervals, and maximum diameter of maximum cross-sectional area was measured (Y [mm]), (Fig. 3c). After paraffin-embedding, cutting to 4-µm slices, hematoxylin/eosin staining, and glass mounting, corresponding site was measured (Y′ [mm]) (Fig. 3d). The shrinkage rate was calculated as (Y − Y′)/Y × 100 (%). From the shrinking rates (1 and 2), the specimen shrinkage rate during the histopathological specimen preparation was evaluated, and a formula to calculate c-DOI from p-DOI was developed. All procedures were performed by a single pathologist.

### Ultrasound measurements

**Figure 4:**
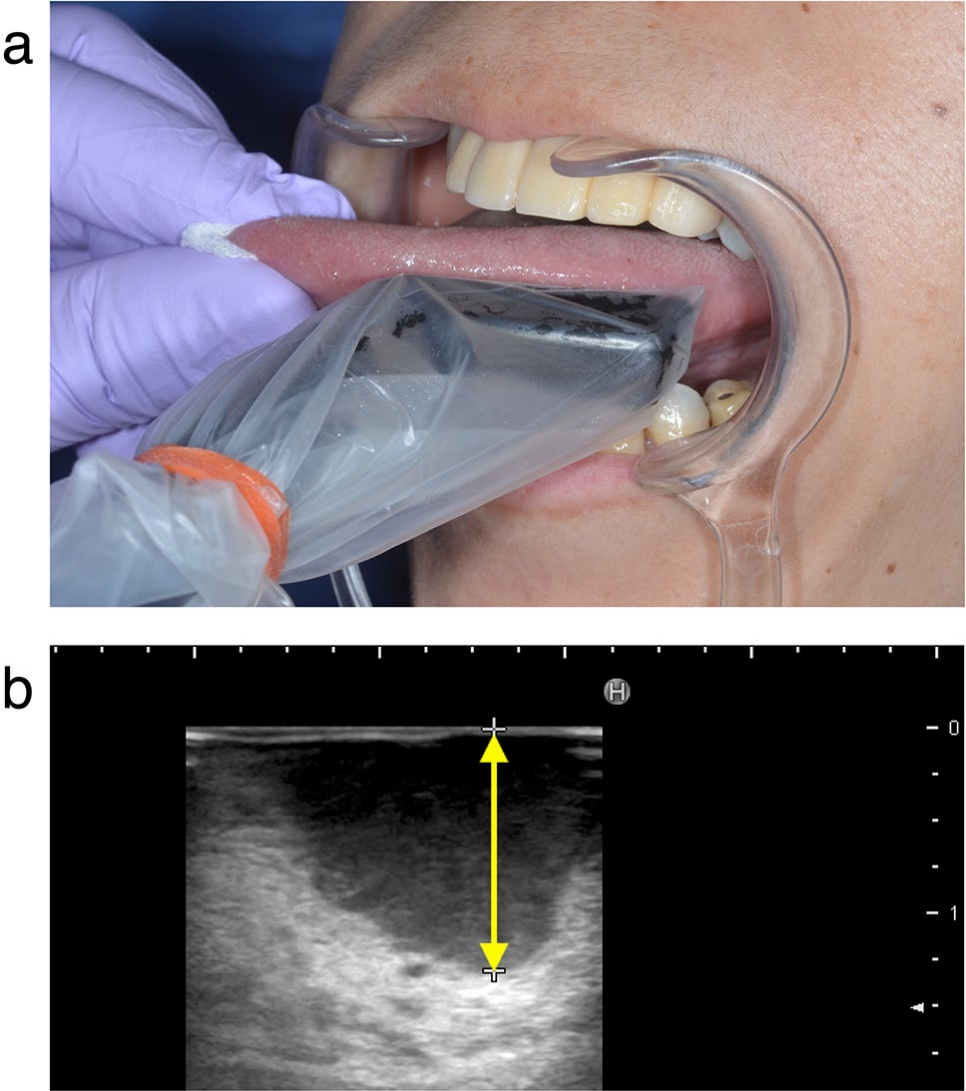
(**a**) Detection of the deepest portion of the tumor. Tongue was let to put forward and measured while holding its apex with gauze. (**b**) Measurement of r-DOI by ultrasound. The tumor was abnormally hypoechoic and the deepest point of tumor was determined. *r-DOI* radiological depth of invasion.

Intraoral ultrasound was performed using an ultrasound unit (HI VISION Avius, Hitachi Healthcare Systems, Japan) with 13-MHz hockey-stick type micro-linear probe. The ultrasound transducer was sheathed with sterile probe cover, with ultrasound gel placed inside the probe cover against the transducer end. Tongue was put forward and measured while holding the tongue’s apex with gauze (Fig. 4a). Xylocaine gel (2%) was applied to the tumor surface. The tumor was abnormally hypoechoic and distorted normal tongue architecture. Real-time scanning of the tumor was performed to determine the deepest point of tumor (Fig. 4b). Using B-mode sonography, we obtained the planes parallel to the tongue’s long axis, with the transducer oriented perpendicular to the deepest portion of the tumor, and r-DOI was measured by oral surgeon with > 10 years of experience.

### MRI measurements

**Figure 5:**
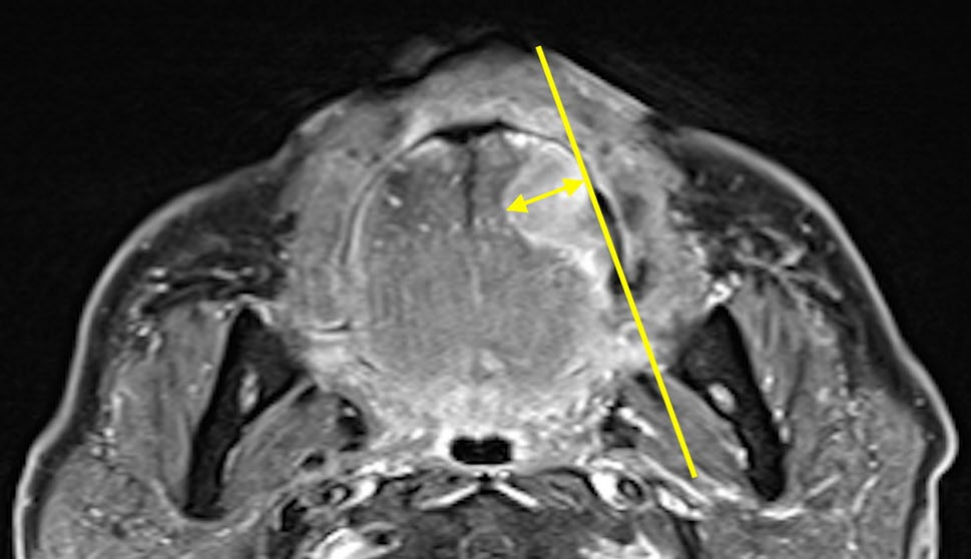
Measurement of r-DOI by MRI. r-DOI using MRI was defined as the vertical distance between the deepest point of the tumor infiltration and the simulated normal mucosal junction. *r-DOI* radiological depth of invasion.

r-DOI using MRI was defined as the vertical distance between the deepest point of the tumor infiltration and the simulated normal mucosal junction^7^ (Fig. 5). In all cases without contraindications for MRI contrast material, r-DOI was measured retrospectively on the axial post-contrast T1-weighted images. MRI was performed on either 1.5 T or 3 T MRI unit (Magnetom Vision or Spectra, Siemens Healthcare, Erlangen, Germany) with a head and neck coil. The scanning protocol included axial and coronal T1-weighted images [TR/TE 500/14 (1.5 T) or 650/10 ms (3 T)], and axial and coronal T2-weighted images with fat suppression (TR/TE 3000/90 or 5000/94 ms). After intravenous injection of contrast material, axial and coronal T1-weighted imaging (TR/TE 640/12 or 500/14 ms) with fat suppression were also performed. All MR images were obtained with a section thickness 3.0–4.0 mm and an intersection gap of 0.9–1.0 mm. The MRI-determined r-DOI was measured by an oral surgeon and radiologist with > 15 years of experience.

### p-DOI measurements

p-DOI of the maximal cross-sectional area of the tumor was measured by an oral surgeon and pathologist with > 15 years of experience who was blinded to the radiological imaging details. p-DOI was measured from the level of the adjacent normal mucosa to the deepest point of tumor infiltration. There were no margin-positive cases in the deepest point.

### Statistical analysis

The correlation between c-DOI and r-DOI measured by ultrasound, MRI before biopsy, and MRI after biopsy was drawn using scatter plots. SPSS version 25 for Windows (SPSS Japan Inc., Tokyo, Japan) was used for this analysis. The Pearson’s regression equations, correlation coefficients, and coefficient of determination for ultrasound, MRI before biopsy, and MRI after biopsy were calculated, respectively. A *p* value < 0.05 was considered statistically significant.

## Supplementary Information

Supplementary Information.

## Data Availability

All data generated or analyzed during this study are included in the published article.

## Abbreviations

DOI: Depth of invasion
c-DOI: Clinical depth of invasion
r-DOI: Radiological depth of invasion
p-DOI: Pathological depth of invasion
TT: Tumor thickness
SCC: Squamous cell carcinoma
