# Supplementary material for: MRI before biopsy correlates with depth of invasion corrected for shrinkage rate of the histopathological specimen in tongue carcinoma
Source: Sci Rep. 2021 Oct 25;11:20992. doi: 10.1038/s41598-021-00398-0 (PMC8545943; doi:10.1038/s41598-021-00398-0)
Supplement: Supplementary file 1 — Supplementary Information. [file 41598_2021_398_MOESM1_ESM.pdf]

MRI before biopsy correlates with depth of invasion corrected for shrinkage rate of the histopathological specimen in tongue carcinoma

Hiroyuki Harada[1*] · Hirofumi Tomioka[1] · Hideaki Hirai[1] · Takeshi Kuroshima[1] · Yu Oikawa[1] · Hitomi Nojima[1] · Junichiro Sakamoto[2] · Tohru Kurabayashi[2] · Kou Kayamori[3] · Tohru Ikeda[3]

[1] Department of Oral and Maxillofacial Surgery, Division of Oral Health Sciences, Tokyo Medical and Dental University, Tokyo, Japan
[2] Department of Oral and Maxillofacial Radiology, Division of Oral Health Sciences, Tokyo Medical and Dental University, Tokyo, Japan
[3] Department of Oral Pathology, Division of Oral Health Sciences, Tokyo Medical and Dental University, Tokyo, Japan

***Corresponding author:**
Hiroyuki Harada, DDS, PhD
Department of Oral and Maxillofacial Surgery, Tokyo Medical and Dental University
1-5-45 Yushima, Bunkyo-ku, Tokyo 113-8549, Japan
Phone No: +81-3-5803-5506
Fax No: +81-3-5803-0199
Email Address: hiro-harada.osur@tmd.ac.jp

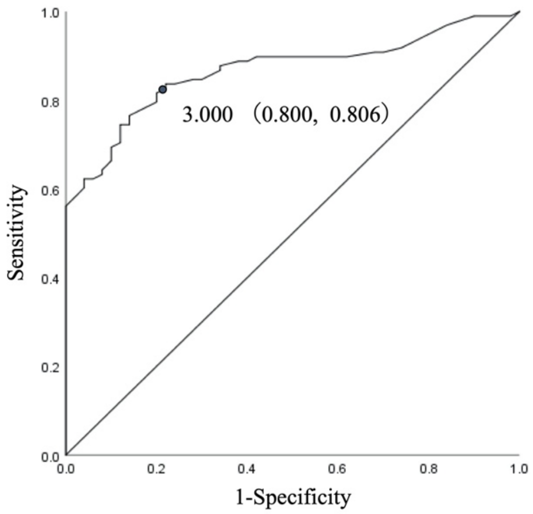

Supplemental Figure S1. Receiver operating characteristic (ROC) curves for detectable and undetectable lesions on MRI showed the results of a 3-mm cut-off (sensitivity 80.6%, specificity 80.0%, and AUC 0.867).

	Before formalin fixation (X mm)	After formalin fixation (X' mm)	Sliced specimen (Y mm)	Slide specimen (Y' mm)	Shrinkage rate by formalin fixation (X→X')%	Shrinkage rate by the preparation process to slide (Y→Y')%
No.1	16.1	15.5	15.5	15.5	3.7	0.0
No.2	10.4	10.2	10.4	9.9	1.9	4.8
No.3	19.2	19.4	19.7	18.0	-1.0	8.6
No.4	11.4	11.8	11.8	10.5	-3.5	11.0
No.5	30.5	32.0	33.0	29.0	-4.9	12.1
No.6	10.0	9.5	9.7	9.7	5.0	0.0
No.7	6.0	6.2	6.3	6.0	-3.3	4.8
No.8	11.5	12.0	11.2	10.9	-4.3	2.7
No.9	12.4	11.7	12.2	10.5	5.6	13.9
No.10	7.0	6.9	6.8	6.0	1.4	11.8
No.11	24.4	23.9	22.2	22.0	2.0	0.9
No.12	19.5	19.4	19.4	18.0	0.5	7.2
No.13	17.7	17.8	17.7	16.7	-0.6	5.6
No.14	12.1	11.3	10.8	9.1	6.6	15.7
No.15	15.9	16.3	16.4	14.0	-2.5	14.6
No.16	18.4	17.9	18.0	16.0	2.7	11.1
No.17	11.1	10.4	10.8	9.9	6.3	8.3
No.18	8.9	8.5	8.3	6.9	4.5	16.9
No.19	7.2	7.0	6.8	6.2	2.8	8.8
No.20	10.6	10.5	10.3	8.8	0.9	14.6
No.21	9.5	9.2	9.4	9.4	3.2	0.0
No.22	10.7	10.7	10.7	9.0	0.0	15.9
No.23	10.3	10.0	10.5	9.2	2.9	12.4
No.24	13.7	13.0	13.1	11.5	5.1	12.2
No.25	16.3	15.5	15.7	14.7	4.9	6.4
Mean ± SD					1.6 ± 3.4	8.8 ± 5.4

Supplemental Table S1. Shrinkage rate during the preparation process of histological specimens.
